# The optimal second-line therapy for older adults with type 2 diabetes mellitus: protocol for a systematic review and network meta-analysis using individual participant data (IPD)

**DOI:** 10.1186/s13643-024-02558-5

**Published:** 2024-06-13

**Authors:** Jingya Wang, Krishnarajah Nirantharakumar, Christopher Sainsbury, David J. Moore, Alan Sinclair, G. Neil Thomas, Wasim Hanif, Megha Singh, Luyuan Tan, Zhaonan Wang, Nikita Simms-Williams, Mi Yao, M. Niluka Gunathilaka, Pushpa Singh, Konstantinos Toulis, Apostolos Tsapas, Dyuti Coomar, Malcolm James Price

**Affiliations:** 1 Institute of Applied Health Research, University of Birmingham, Birmingham, UK; 2 Midlands Health Data Research UK, Birmingham, UK; 3 Foundation for Diabetes Research in Older People, King’s College London, London, UK; 4 Department of Diabetes and Endocrinology, University Hospital Birmingham, Birmingham, UK; 5 Department of General Practice, Peking University First Hospital, Beijing, China; 6 Institute of Metabolism and Systems Research, University of Birmingham, Birmingham, UK; 7 Department of Endocrinology, General Military Hospital, Thessaloniki, Greece; 8 Diabetes Centre, Second Medical Department, Ippokratio General Hospital, Thessaloniki, Greece; 9 Harris Manchester College, University of Oxford, Oxford, UK; 10 Clinical Research and Evidence-Based Medicine Unit, Second Medical Department, Aristotle University of Thessaloniki, Thessaloniki, Greece

**Keywords:** Randomised controlled trials, Glucose-lowering agents, Glucagon-like peptide-1 agonists, Sodium-glucose cotransporter-2 inhibitors, Dipeptidyl peptidase-4 inhibitors, Biguanide, Sulfonylureas, Thiazolidinediones, Metformin, Insulin

## Abstract

**Background:**

Due to increasing life expectancy, almost half of people with type 2 diabetes are aged 65 years or over worldwide. When metformin alone does not control blood sugar, the choice of which second-line therapy to prescribe next is not clear from currently available evidence. The existence of frailty and comorbidities in older adults further increases the complexity of medical decision-making. As only a relatively small proportion of trials report results separately for older adults, the relative efficacy and safety of second-line therapies in older adults with type 2 diabetes mellitus are unknown and require further investigation. This individual participant data (IPD) network meta-analysis evaluates the relative efficacy and safety of second-line therapies on their own or in combination in older adults with type 2 diabetes mellitus.

**Methods:**

All relevant published and unpublished trials will be identified. Studies published prior to 2015 will be identified from two previous comprehensive aggregate data network meta-analyses. Searches will be conducted in CENTRAL, MEDLINE, and EMBASE from 1st January 2015 onwards, and in clinicaltrials.gov from inception. Randomised controlled trials with at least 100 estimated older adults (≥ 65 years) receiving at least 24 weeks of intervention that assess the effects of glucose-lowering drugs on mortality, glycemia, vascular and other comorbidities outcomes, and quality of life will be eligible. The screening and data extraction process will be conducted independently by two researchers. The quality of studies will be assessed using the Cochrane risk of bias tool 2. Anonymised IPD of all eligible trials will be requested via clinical trial portals or by contacting the principal investigators or sponsors. Received data will be reanalysed where necessary to standardise outcome metrics. Network meta-analyses will be performed to determine the relative effectiveness of therapies.

**Discussion:**

With the increasing number of older adults with type 2 diabetes worldwide, an IPD network meta-analysis using data from all eligible trials will provide new insights into the optimal choices of second-line antidiabetic drugs to improve patient management and reduce unnecessary adverse events and the subsequent risk of comorbidities in older adults.

**Systematic review registration:**

PROSPERO CRD42021272686.

**Supplementary Information:**

The online version contains supplementary material available at 10.1186/s13643-024-02558-5.

## Background

Changes in global demographics and increased life expectancy have led to a greater prevalence of type 2 diabetes mellitus (T2DM) in older adults, who will benefit from high-quality diabetes care to minimise diabetes-related complications [1, 2]. Globally, 135 million older adults aged 65–99 years were affected by T2DM in 2019 [3]. This number is expected to reach 195 million by 2030 and 276 million by 2045 [3]. In England, over half of individuals with T2DM were aged 65 or over in 2021 [4].

The prevalence of frailty and comorbidities in older adults increases the complexity of medical decision-making [5]. Older adults have an inherently higher cardiovascular risk, higher levels of cognitive impairment, and a higher prevalence of chronic kidney disease (CKD) than younger people. There is also increased potential for adverse outcomes from the side effects of medication (e.g. hypoglycaemia and osteoporosis with sulphonylureas and pioglitazone) [6]. Therefore, medication must be targeted for relative risk reduction of adverse events as well as additional comorbidities whilst considering potential drug-drug interactions, which may constrain the available choices of therapeutic agents [7–9].

Although older adults are the majority group in clinical practice, they are an underserved population within existing clinical trials [10]. In the most up-to-date systematic review on antidiabetic drugs, only 7 out of 453 included trials focused on this age group, and outcomes for this group are seldom reported separately [11]. Current knowledge and previous guidelines for T2DM have been largely based on randomised controlled trials (RCTs) in which the majority of participants are less than 65 years old [12]. The recently released guidelines on diabetes in older adults from the American Diabetes Association (ADA) and European Society of Endocrinology (ESE) provide a comprehensive account of management, but they do not give evidence-based decision-making guidance on the choice of second-line therapy [13, 14].

Older adults are a heterogeneous group compared with younger adults in terms of pharmacokinetics and pharmacodynamics due to a greater degree of comorbidity including age-related renal dysfunction, cardiovascular disease, and other physiological disturbances, and the presence of sarcopenic obesity and frailty [1]. Therefore, a clear understanding of the treatment effect is important if optimum therapy is to be chosen. Similarly, the study of the impact of glycaemic control has demonstrated that the benefits of tight glycaemic control in older adults are minimal and that there is a U-shaped relationship between chronic glycaemic control (HbA1c) and adverse outcomes [15]. In the new quality outcome framework for older adults, there is more emphasis on deintensification of treatment, with less tight glycaemic targets [16].

Metformin is the recommended first-line therapy globally, but there is no clear consensus regarding second-line therapy (the next agent to choose to add to metformin at the point of treatment intensification) in older adults with T2DM. Available evidence suggests that the relationships between therapy and outcome may differ between older adults and younger people [17]. Therefore, the common approach of recruiting people with a wide range of ages to trials without age stratification and subgroup/regression analysis does not fully describe the response in this older group [15, 18]. They are an underserved population within existing clinical trials (despite representing the majority of individuals with diabetes in high-income countries), leading to a lower level of understanding of how this important group responds to therapy. This population has the most to gain (reduction in mortality, morbidity and consequently increased quality of life) from optimal therapy choice, and the most to lose from side effects or lost benefit from suboptimal therapy.

Hence, the aim of this study is to perform a systematic review and network meta-analysis (NMA) using individual participant data (IPD) from all relevant eligible randomised controlled trials to identify the best second-line antidiabetic therapy option for older adults with T2DM.

## Methods/design

This study will be a systematic review and NMA using IPD. This protocol adheres to Preferred Reporting Items for Systematic review and Meta-Analysis Protocols (PRISMA-P) [19] and guidelines for NMA protocols [20]. The PRISMA for Individual Patient Data Systematic reviews (PRISMA-IPD) [21] and PRISMA for Network Meta-Analyses (PRISMA-NMA) [22] will be followed when reporting the findings of the study.

### Patient and public involvement (PPI)

This protocol has been developed in consultation with older adults with T2DM and their carers, as well as with NHS clinicians who routinely care for individuals with T2DM in primary care settings. The patient group will be regularly consulted as the research progresses via focus group discussions.

### Aim

1. To evaluate the relative efficacy and safety of second-line therapies on their own or in combination in older adults with T2DM.
2. To compare the differential relative effectiveness of second-line treatment between those aged over and below 65 years of age.

### Design

#### Types of studies

RCTs with either a parallel arm or cross-over design will be eligible. For trials with a cross-over design, only the information from the first stage will be used. Ongoing trials will be excluded from this study but will be listed for future reference.

#### Trial participants

Eligible participants will be individuals with T2DM. If the number of older adults (≥ 65 years) is not provided in the trial report, the number of older adults will be estimated from the study sample size, mean, and standard deviation. Given that our primary objective is to evaluate the performance of second-line therapies in older adults with T2DM, trials with fewer than 100 estimated older adults will be excluded.

### Types of interventions and comparators

Eligible treatments include drugs with a primary indication to lower blood glucose that have been approved or have applied for marketing authorisation either by the U.S. Food and Drug Administration (FDA) or the European Medicines Agency (EMA), as of December 2020. The following drug classes are considered: biguanides, sulfonylureas, thiazolidinediones, dipeptidyl peptidase-4 (DPP-4) inhibitors, sodium-glucose cotransporter-2 (SGLT-2) inhibitors, glucagon-like peptide-1 (GLP-1) receptor agonists, insulins, meglitinides, and alpha-glucosidase inhibitors.

Trials comparing an eligible intervention of one drug class with another eligible intervention of a different drug class or placebo or standard therapy or no treatment (interclass comparison) will be included. For GLP-1 agonists and SGLT-2 inhibitors, trials comparing a GLP-1 agonist or an SGLT-2 inhibitor with another eligible intervention of the same drug class (intraclass comparison) will also be included. Monotherapy, dual, or triple combinations of eligible medications will be included.

Where trials have background therapy, this must be similar across randomised groups. Eligible background therapy can be any glucose-lowering medication throughout the intervention period.

As the focus of this study is second-line therapy, trials comparing the first-line therapy (metformin) and placebo only will be excluded. The duration of treatment during the randomised period (or first period for cross-over trials) must be at least 24 weeks to ensure the full effect of the treatment can be observed and to avoid potential reverse causation.

### Types of outcome measures

The primary outcomes are HbA1c level and all-cause mortality. Secondary efficacy and safety outcomes include myocardial infarction, stroke, heart failure, cardiovascular mortality, body weight, low-density lipoprotein cholesterol/dyslipidaemia, blood pressure/hypertension, hypoglycaemia, kidney diseases, liver diseases, diabetic retinopathy, diabetic foot diseases/amputation, diabetic ketoacidosis, quality of life, physical performance, frailty, patient-reported outcomes, and hospitalisation. Eligible trials must report at least one of the outcomes above. If the same outcome was measured multiple times between 24 weeks and the end of the study follow-up, all outcome data measured during the period of receipt of intervention/comparator will be analysed if possible.

### Information sources and search strategy

Literature searches to identify published and unpublished trials will build on two previous comprehensive aggregate data NMAs [11, 23]. Both examined antidiabetic therapies for individuals with T2DM without age restriction. They searched for trials up to 1st March 2016 [23] and 29th September 2020 [11] respectively. Whilst the inclusion and exclusion criteria are closely related to this protocol, there are differences in scope. Therefore, the following strategy is used to maintain a sensitive and specific search without returning an overwhelming yield of records to screen:

1. Cochrane Central Register of Controlled Trials (CENTRAL), MEDLINE, and EMBASE will be searched from 1st January 2015 onwards without language or location restriction. The search terms are described in the Supplementary file 1 and PROSPERO registration [24];
2. Clinicaltrials.gov and the International Clinical Trials Registry Platform (ICTRP) will be searched from inception;
3. The list of trials excluded at full-text screening together with reasons from the previous NMAs will be requested from the authors.
4. Reference lists of eligible trials and the most relevant systematic reviews identified in our database searches (point 1) will be checked.

Deidentified IPD for included trials will be requested via three major clinical trial portals (Vivli [25], Clinical Study Data Request [CSDR] [26], and the Yale University Open Data Access [YODA] [27]). For trials that are not available on these portals, the principal investigators or sponsors will be contacted to request the IPD.

### Data management and selection process

**Fig. 1:**
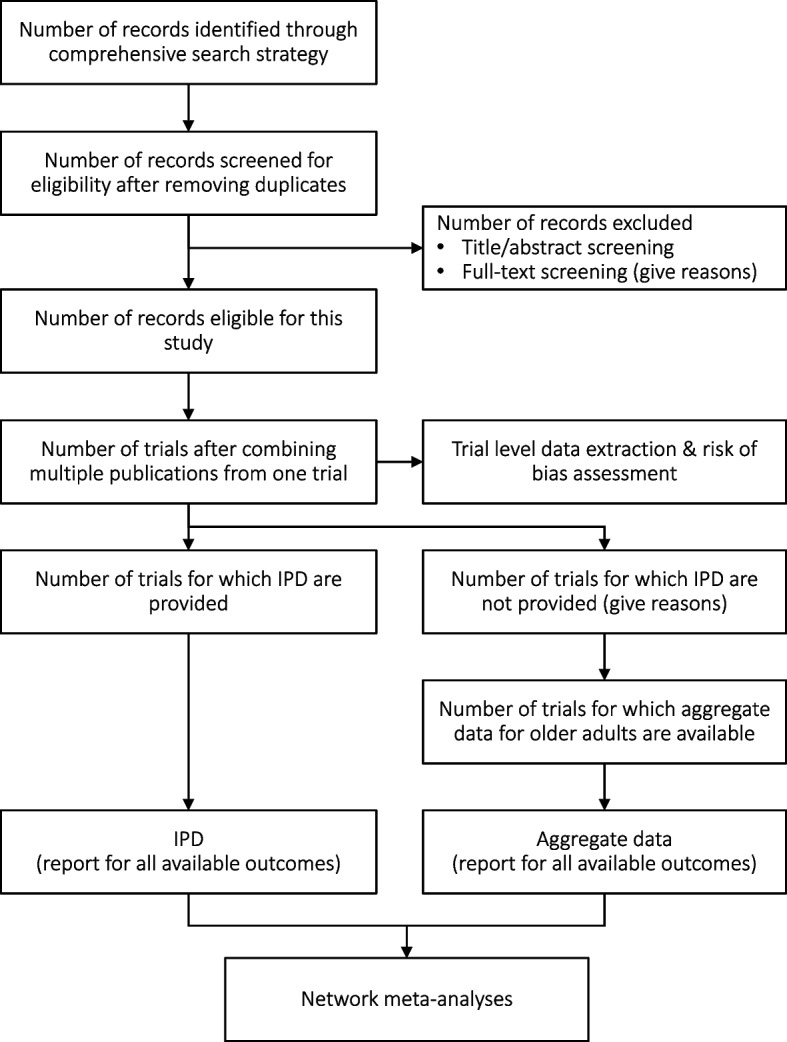
Flow diagram

Study records will be kept and managed using EndNote (X20, Clarivate Analytics) bibliographic software. Duplicate records will be removed automatically and manually. Titles and abstracts will be screened for relevance to the review aims by one reviewer. A second reviewer will check a random selection of 10% of the records, and inter-rater reliability will be calculated. Full copies of relevant articles will be obtained and assessed against the full selection criteria by two of the five listed reviewers (MS, LT, ZW, NW and/or MY) independently. Any conflicts will be discussed and, if need be, resolved through a third reviewer (JW). Reasons for exclusion of studies at this stage will be recorded. The selection process will be reported using a PRISMA-IPD flow diagram [28] Fig. 1.

### Data collection process and data items

#### Study level data

An adapted Cochrane RCT data collection form [29] will be used to extract study-level data for each eligible trial, including the following domains: trial characteristics (trial registration number, trial name, aim, design, year, language and sponsor), participants (eligibility criteria, sample size, baseline age, subgroup of older adults, and background therapies), intervention and comparison groups (drug information and treatment duration) and outcome (definition and time of measurement). Data extraction will be conducted at the trial level by two of the five listed reviewers (MS, LT, ZW, NW, and/or MY) independently and checked by the third person (JW). For trials with multiple publications, information across all of them will be checked. If available, trial protocols and technical reports will be checked for additional information and consistency with the publications.

#### Participant level data

All obtained IPD will be kept, managed, and processed in secure research environments as per requirements from principal investigators, sponsors of trials, trial portals, the National Institute for Health Research (NIHR), and the University of Birmingham Data Management Policy. Only authorised team members will have access to these data.

Trials for which IPDs are not available or not obtained will be listed along with the reasons. The study characteristics of these trials will be compared to those where IPD is obtained.

The anonymised IPD requested from each trial will include the following:

1. Study design information: the date of randomisation, dates of follow-up visits, antidiabetic drug information, and adherence data;
2. Baseline demographic characteristics: age, sex, ethnicity, education and deprivation information, weight, height, smoking, and alcohol consumption;
3. Baseline morbidities: diabetes duration, diabetic complications, hypertension, dyslipidaemia, atrial fibrillation, ischemic heart disease, stroke or transient ischemic attack, rheumatoid arthritis, chronic respiratory disease, cancer, depression, anxiety, cognitive disability;
4. Baseline biomarkers: HbA1c, systolic/diastolic blood pressures, lipid profile, and kidney function indicators;
5. Baseline concurrent prescriptions: antidiabetic drugs; antihypertensive drugs, lipid-lowering drugs, antiplatelets, and anticoagulants;
6. Outcomes: relevant outcomes recorded at each time of measurement.

### IPD integrity

The imbalance between arms within the older adult group will be assessed in each trial by reporting baseline characteristics for key demographic and clinical variables, as described in statistical methods below.

### Risk of bias in individual studies

Risk of bias (RoB) for included trials will be assessed and reported using the Cochrane Risk of Bias tool 2, with outcome-specific assessment only undertaken for the primary outcomes of the review [30].

### Synthesis methods

A two-stage analysis will be undertaken. In the first stage, each trial will be analysed independently to estimate adjusted treatment effects and associated standard errors for each of the primary and secondary outcomes recorded by the trial. In the second stage, a random-effects NMA will be performed to synthesise the results for each outcome.

#### Stage 1—independent analysis of the data from each trial

In the first stage, relevant parameters will be estimated independently for each outcome in each trial.

The baseline characteristics for each trial, stratified by arm, will be summarised. These will include outcomes measured at baseline (e.g. HbA1c), demographic variables such as age and sex, co-morbidities, and other prognostic factors. Binary/categorical variables will be reported as numbers and percentages in each category. Continuous variables will be summarised using the mean and standard deviation if they have an approximately symmetrical distribution, or median and interquartile range otherwise.

Estimation of treatment effects will be on an intention-to-treat basis using multivariable regression modelling. For continuous outcomes reported at follow-up only, a linear regression model will be fitted. For continuous outcomes measured at baseline and follow-up, adjustment for baseline values will be performed using a linear regression model (analysis of covariance). For binary outcomes, a log-linear model will be fitted to estimate risk ratios (RRs). If significant issues with model convergence are encountered, a logistic regression model will be fitted instead, using the Firth method to accommodate rare outcomes [31]. Time-to-event outcomes (e.g. all-cause mortality) will be analysed using Cox regression. Kaplan–Meier curves and log–log plots will be assessed to determine whether the proportional hazards assumption appears reasonable. If any of the outcomes are ordinal (e.g. perhaps physical performance), an ordinal logistic regression model will be fitted. The linear predictor in all models will include an intercept, treatment parameter, and parameters for prognostic factors. For the analysis of cluster trials, mixed-effects multivariable regression models with a random intercept across clusters will be used.

All trial participants aged 65 or over will be included in the primary analysis. Planned subgroup analysis will only include participants relevant to the subgroup (e.g. those with pre-existing cardiovascular disease). The exploration of variation of effects section below gives more detail on planned subgroup analyses.

In general, the authors’ definitions of the outcomes will be used. However, where possible, outcome measures will be converted to the same scale (e.g. HbA1c and HbA1c%). Where this is not possible, standardised mean differences will be considered.

Patients with missing outcome data will be excluded from the analysis for that outcome. Any prognostic factors that are not reported by most included trials will not be considered. For prognostic factors with less than 5% missing data in a given trial, a complete case analysis will be performed. Otherwise, data will be imputed using multiple imputations.

The relevant results will be exported into a single data file ready for the stage 2 analysis. These will include a summary of treatment effects and standard errors for each trial. For multi-arm trials, the relevant correlation coefficients from the correlation matrix for the regression model will also be exported. This will be done for overall results and results from subgroup analyses (e.g. patients with a given co-morbidity). Articles reporting results for eligible studies for which IPD could not be attained will be checked for any results stratified by age. If sufficient information is reported, the appropriate summary results will be extracted and incorporated into the analyses.

#### Stage 2—NMA to synthesise results across trials

In stage 2, a random-effects NMA model will be fitted to jointly synthesize the results from all the included trials [32]. The primary analysis will not account for drug dose. A multivariate meta-regression model fit using restricted maximum likelihood estimation (REML) will be implemented to perform the NMA. The method accounts for the correlation between results from multi-arm trials under the assumption of a multivariate Normal distribution for the treatment effects (or the natural log of the treatment effects) both within and between trials. The most commonly studied treatment will be chosen as the reference treatment to minimize data augmentation in the frequentist framework. A common between-study variance parameter across the different treatment contrasts will be assumed. The NMA will use the effect estimates and standard errors (on the natural log scale for RRs, ORs and HRs) and correlation coefficients for multi-arm trials calculated in stage 1.

The assumption of transitivity will be assessed epidemiologically by considering the distributions of covariates that are potential effect modifiers across trials using graphical displays. Statistical tests will be used to assess evidence for global (using the design-by-treatment interaction model) [33] and local (using node-splitting) inconsistency in the NMA [34].

Summary treatment effects will be reported for all treatment contrasts. The relative treatment effects (all-adjusted) for dichotomous outcomes will be summarised as RRs; ordinal outcomes as odds ratios (ORs); continuous outcomes as mean differences (MDs) or standardized mean differences (SMDs) if appropriate; and time-to-event outcomes as hazard ratios (HRs) [35]. Confidence intervals (95%) will be reported. These summary treatment effects, together with estimates based on direct evidence only, and indirect evidence only, will be reported in forest plots.

Heterogeneity will be assessed using the *I*^2^ statistic for each pairwise comparison [33]. The common between-study variance from the NMA will be reported, and the value compared to the empirical distribution of between-study variance estimates calculated by Turner et al. [36].

The probability that each treatment is of each rank will be calculated using resampling methods [37]. The surface under the cumulative ranking curve (SUCRA) [38], and the mean ranks and quantile ranks will be reported for each treatment. For primary outcomes, the percentage contribution of each trial and the Borrowing of Strength (BoS) statistic for each treatment contrast will be reported [39]. Network diagrams will be presented together with other appropriate graphs such as extended forest plots of summary estimates and rankograms. All analyses will be conducted in R 4.3.0 (R Foundation for Statistical Computing).

It is recognised that IPD meta-analyses are challenging. If changes to the analysis plan are made, these will be clearly reported. Any post-hoc analyses will be clearly labelled and indicated as being only hypothesis-generating. If other nuances not envisaged occur, the recommendations set out in Riley et al. [32] will be followed where possible.

### Exploration of variation in effects

The following pre-specified sub-group analyses will be performed:

Splitting the comparisons into:

- Monotherapy only
- Dual therapy only
- Triple therapy only

Following comprehensive discussions with our expert panel, the following pre-specified sub-group analyses will be performed:

- Age
- Follow-up time
- Sex
- Body mass index
- Presence of cardiovascular disease at baseline
- Presence of CKD at baseline.

### Risk of bias across studies

The reasons given by data holders for not providing data for any study from which it was requested will be reported. Sensitivity analyses will be performed, removing all studies from sponsors from whom data that were requested were not received, and without a clear reason that was independent of the study results. For example, if companies clearly state a policy for data sharing of all trials only after a certain time point, then these studies will not be the subject of sensitivity analysis.

### Additional analyses

Sensitivity analyses using only data from studies judged to be at low risk of bias will be performed.

### Meta-biases

The potential for small-study effects will be assessed by producing comparison-adjusted funnel plots [40].

## Discussion

This will be the first systematic review and IPD NMA focusing on the optimal second-line therapy for older adults with T2DM. However, several potential limitations should be acknowledged. The clinical trial data-sharing commitment was only endorsed among members of the Pharmaceutical Research and Manufacturers of America (PhRMA) and European Federation of Pharmaceutical Industries and Associations (EFPIA) in 2013 [41], so individual-level data before this point will likely be more difficult to obtain, the quality and availability of individual participant data from eligible trials may vary, which may introduce heterogeneity and potential biases into the analyses. There may be a risk of publication bias/accessibility bias, where trials with positive or significant results are more likely to be published/accessed than those without, leading to an overestimation of treatment effects. In light of this, all declined reasons for data requests will be documented. The trial-level characteristics will be compared according to whether IPD was available, the trial start year, data sources, and the result of the RoB assessment. Because results for older adults are rarely reported separately in publications, common methods to assess these biases such as funnel plots may not be feasible.

Given the large number of older adults with T2DM now and in the future, together with the potentially sufficient existing body of evidence hidden in previous trials, the findings from this study could ultimately be used to guide clinical decisions about the optimal second-line treatment in terms of efficacy and safety for older adults with T2DM. This is of immense importance to patients, carers, healthcare professionals, policymakers, and researchers. The results will be published in peer-reviewed journals. Findings will also be made available in formats accessible to patient and carer groups, healthcare professionals, policymakers, researchers, relevant charities, and other stakeholders.

### Supplementary Information

Supplementary Material 1. MEDLINE search (1946 to November Week 4 2020) Search Date: 3 Dec 2020.

## Data Availability

All relevant data and materials have been provided in the manuscript.

## Abbreviations

IPD: Individual Participant Data
T2DM: Type 2 diabetes mellitus
CKD: Chronic kidney disease
RCTs: Randomised controlled trials
ADA: American Diabetes Association
ESE: European Society of Endocrinology
HbA1c: Haemoglobin A1c
NMA: Network Meta-Analysis
PRISMA-P: Preferred Reporting Items for Systematic review and Meta-Analysis Protocols
FDA: U.S. Food and Drug Administration
EMA: European Medicines Agency
DPP-4: Dipeptidyl peptidase-4
SGLT-2: Sodium-glucose cotransporter-2
GLP-1: Glucagon-like peptide-1
CENTRAL: Cochrane central register of controlled trials
CSDR: Clinical Study Data Request
YODA: Yale University Open Data Access
NIHR: National Institute for Health Research
RoB: Risk of bias
ORs: Odds ratios
MDs: Mean differences
SMDs: Standardised mean differences
HRs: Hazard ratios
SUCRA: Surface under the cumulative ranking curve
BoS: Borrowing of strength
