# Supplementary material for: The optimal second-line therapy for older adults with type 2 diabetes mellitus: protocol for a systematic review and network meta-analysis using individual participant data (IPD)
Source: Syst Rev. 2024 Jun 13;13:155. doi: 10.1186/s13643-024-02558-5 (PMC11170882; doi:10.1186/s13643-024-02558-5)
Supplement: Supplementary file 1 — Supplementary Material 1. MEDLINE search (1946 to November Week 4 2020) Search Date: 3 Dec 2020. [file 13643_2024_2558_MOESM1_ESM.docx]

**MEDLINE search (1946 to November Week 4 2020) Search Date: 3 Dec 2020**

1. exp Diabetes Mellitus Type 2/ or Diabetes Mellitus/

2. non insulin dependent diabetes mellitus/

3. ((diabetes or diabetes mellitus or diabetic*) adj1 (type 2 or type II or type ii or non-insulin dependent or noninsulin dependent or adult onset or mature onset or late onset)).tw.

4. NIDDM.tw.

5. Diabetic Nephropathies/

6. (diabetic nephropath* or diabetic kidney disease).tw.

7. 1 or 2 or 3 or 4 or 5 or 6

8. Insulin/ or Insulins/ or Insulin Aspart/ or Insulin Detemir/ or Insulin Glargine/ or Insulin, Isophane/ or Insulin Lispro/ or Insulin, Regular, Human/ or Insulin, Lente/ or Isophane Insulin, Human/ or Insulin, Ultralente/ or exp Insulin, Long Acting/

9. ((insulin adj1 aspart) or (insulin adj1 detemir) or (insulin adj1 glargine) or (insulin adj1 isophane) or (insulin adj1 lispro) or ((long acting or longer acting or intermediate acting) adj insulin*) or (insulin adj1 lente) or (insulin adj1 ultralente)).tw.

10. (insulin adj1 degludec).tw.

11. (insulin adj1 zinc).tw.

12. (insulin adj1 glulisine).tw.

13. Biphasic Insulins/

14. (novorapid or fiasp).mp.

15. Biguanides/ or biguanides.tw. or Metformin/ or metformin.tw.

16. alpha-Glucosidases/ or alpha glucosidase inhibitor*.tw.

17. Acarbose/ or acarbose.tw.

18. miglitol.tw.

19. voglibose.tw.

20. Amylin Receptor Agonists/ or (amylin adj1 (analogue* or derivative*)).tw. or pramlintide.tw.

21. meglitinide*.tw.

22. mitiglinide.tw.

23. Nateglinide/ or nateglinide.tw.

24. repaglinide.tw.

25. Thiazolidinediones/ or thiazolidinedione*.tw.

26. glitazone*.tw.

27. Pioglitazone/ or pioglitazone.tw.

28. rivoglitazone.tw.

29. Rosiglitazone/ or rosiglitazone.tw.

30. lobeglitazone.tw.

31. Receptors, Glucagon/ag or Glucagon-Like Peptide-1 Receptor/ or Glucagon-Like Peptide 1/ag

32. (glucagon-like peptide 1 receptor inhibitor* or glucagon-like peptide 1 receptor agonist* or glucagon-like peptide 1 inhibitor* or glucagon-like peptide 1 agonist* or GLP-1 receptor inhibitor* or GLP-1 receptor agonist* or GLP-1 inhibitor* or GLP-1 agonist*).tw.

33. albiglutide.tw.

34. dulaglutide.tw.

35. (exenatide or exendin 4).tw.

36. Liraglutide/ or liraglutide.tw.

37. lixisenatide.tw.

38. semaglutide.tw.

39. taspoglutide.tw.

40. Sodium-Glucose Transporter 2 Inhibitors/ or (sodium glucose transporter 2 inhibitor* or sodium glucose transporter ii inhibitor* or SGLT 2 inhibitor* or (sodium glucose cotransporter adj3 inhibitor*) or (sodium glucose co transporter adj3 inhibitor*)).tw.

41. Canagliflozin/ or canagliflozin.tw.

42. dapagliflozin.tw.

43. empagliflozin.tw.

44. ertugliflozin.tw.

45. ipragliflozin.tw.

46. luseogliflozin.tw.

47. remogliflozin etabonate.tw.

48. sotagliflozin.tw.

49. tofogliflozin.tw.

50. Sulfonylurea Compounds/ or (sulphonylurea or sulphonylureas).tw.

51. Acetohexamide/ or acetohexamide.tw.

52. Carbutamide/ or carbutamide.tw.

53. Chlorpropamide/ or chlorpropamide.tw.

54. glibenclamide.tw.

55. glibornuride.tw.

56. Gliclazide/ or gliclazide.tw.

57. glimepiride.tw.

58. Glipizide/ or glipizide.tw.

59. gliquidone.tw.

60. glisoxepide.tw.

61. Glyburide/ or glyburide.tw.

62. glyclopyramide.tw.

63. glycopyramide.tw.

64. glycyclamide.tw.

65. metahexamide.tw.

66. Tolazamide/ or tolazamide.tw.

67. Tolbutamide/ or tolbutamide.tw.

68. tolcyclamide.tw.

69. Dipeptidyl-Peptidase IV Inhibitors/ or (dipeptidyl-peptidase IV Inhibitor* or dipeptidyl-peptidase 4 Inhibitor* or ((DPP4 or DPP 4 or DPP IV) adj inhibitor*)).tw.

70. alogliptin.tw.

71. anagliptin.tw.

72. dutogliptin.tw.

73. evogliptin.tw.

74. gemigliptin.tw.

75. gosogliptin.tw.

76. Linagliptin/ or linagliptin.tw.

77. omarigliptin.tw.

78. saxagliptin.tw.

79. septagliptin.tw.

80. Sitagliptin Phosphate/ or (sitagliptin or Sitagliptin Phosphate).tw.

81. teneligliptin.tw.

82. trelagliptin.tw.

83. Vildagliptin/ or vildagliptin.tw.

84. Hypoglycemic Agents/

85. or/8-84

86. (randomized controlled trial or controlled clinical trial or pragmatic clinical trial).pt. or randomized.ab. or placebo.ab. or clinical trials as topic/ or randomly.ab. or trial.ti. or randomized.ti. or placebo.ti.

87. animals/ not (humans/ and animals/)

88. 86 not 87

89. 7 and 85 and 88

90. limit 89 to yr="2015 -Current"

**EMBASE search (1974 to 2020 December 02) Search Date: 3 Dec 2020**

1. diabetes mellitus/

2. non insulin dependent diabetes mellitus/

3. ((diabetes or diabetes mellitus or diabetic*) adj1 (type 2 or type II or type ii or non-insulin dependent or noninsulin dependent or adult onset or mature onset or late onset)).tw.

4. NIDDM.tw.

5. Diabetic Nephropathy/

6. (diabetic nephropath* or diabetic kidney disease).tw.

7. or/1-6

8. Insulin/ or insulin glargine/ or insulin degludec/ or insulin zinc suspension/ or biphasic insulin/ or long acting insulin/ or human insulin/ or insulin detemir/ or insulin glulisine/ or insulin aspart/ or insulin lispro/ or isophane insulin/

9. insulin derivative/

10. ((insulin adj1 glargine) or (insulin adj1 degludec) or (insulin adj1 zinc) or ((long acting or longer acting or intermediate acting) adj insulin*) or (insulin adj1 detemir) or (insulin adj1 glulisine) or (insulin adj1 aspart) or (insulin adj1 lispro) or (insulin adj1 isophane) or (insulin adj1 lente) or (insulin adj1 ultralente)).tw.

11. (novorapid or fiasp).mp.

12. biguanide/ or biguanide derivative/ or biguanide*.tw. or metformin/ or metformin.tw,sh.

13. alpha glucosidase inhibitor/ or alpha glucosidase inhibitor*.tw.

14. acarbose/ or acarbose.tw.

15. miglitol/ or miglitol.tw.

16. voglibose/ or voglibose.tw.

17. amylin derivative/ or (amylin adj1 (analogue* or derivative*)).tw. or pramlintide/ or pramlintide.tw.

18. meglitinide/ or meglitinide*.tw.

19. mitiglinide/ or mitiglinide.tw.

20. nateglinide/ or nateglinide.tw.

21. repaglinide/ or repaglinide.tw.

22. 2,4 thiazolidinedione derivative/ or thiazolidinedione*.tw.

23. glitazone derivative/ or (glitazone adj1 (derivative* or analogue*)).tw.

24. pioglitazone/ or pioglitazone.tw.

25. rivoglitazone/ or rivoglitazone.tw.

26. rosiglitazone/ or rosiglitazone.tw.

27. lobeglitazone/ or lobeglitazone.tw.

28. glucagon like peptide 1 receptor agonist/ or (glucagon-like peptide 1 receptor inhibitor* or glucagon-like peptide 1 receptor agonist* or glucagon-like peptide 1 inhibitor* or glucagon-like peptide 1 agonist* or GLP-1 receptor inhibitor* or GLP-1 receptor agonist* or GLP-1 inhibitor* or GLP-1 agonist*).tw.

29. albiglutide/ or albiglutide.tw.

30. dulaglutide/ or dulaglutide.tw.

31. exendin 4/ or (exenatide or exendin 4).tw.

32. liraglutide/ or liraglutide.tw.

33. lixisenatide/ or lixisenatide.tw.

34. semaglutide/ or semaglutide.tw.

35. taspoglutide/ or taspoglutide.tw.

36. sodium glucose cotransporter 2 inhibitor/ or (sodium glucose transporter 2 inhibitor* or sodium glucose transporter ii inhibitor* or SGLT 2 inhibitor* or (sodium glucose cotransporter adj3 inhibitor*) or (sodium glucose co transporter adj3 inhibitor*)).tw.

37. canagliflozin/ or canagliflozin.tw.

38. dapagliflozin/ or dapagliflozin.tw.

39. empagliflozin/ or empagliflozin.tw.

40. ertugliflozin/ or ertugliflozin.tw.

41. ipragliflozin/ or ipragliflozin.tw.

42. luseogliflozin/ or luseogliflozin.tw.

43. remogliflozin etabonate/ or remogliflozin etabonate.tw.

44. sotagliflozin/ or sotagliflozin.tw.

45. tofogliflozin/ or tofogliflozin.tw.

46. sulfonylurea/ or sulfonylurea derivative/ or sulphonylurea derivative/ or sulphonylurea*.tw. or sulfonylurea*.tw.

47. acetohexamide/ or acetohexamide.tw.

48. carbutamide/ or carbutamide.tw.

49. chlorpropamide/ or chlorpropamide.tw.

50. glibenclamide/ or glibenclamide.tw.

51. glibornuride/ or glibornuride.tw.

52. gliclazide/ or gliclazide.tw.

53. glimepiride/ or glimepiride.sh,tw.

54. glipizide/ or glipizide.tw.

55. gliquidone/ or gliquidone.tw.

56. glisoxepide/ or glisoxepide.tw.

57. glyburide.tw.

58. glyclopyramide.tw.

59. glycopyramide.tw.

60. glycyclamide/ or glycyclamide.tw.

61. metahexamide/ or metahexamide.tw.

62. tolazamide/ or tolazamide.tw.

63. tolbutamide/ or tolbutamide.tw.

64. tolcyclamide.tw.

65. dipeptidyl peptidase IV inhibitor/ or (dipeptidyl-peptidase IV Inhibitor* or dipeptidyl-peptidase 4 Inhibitor* or ((DPP4 or DPP 4 or DPP IV) adj inhibitor*)).tw.

66. alogliptin/ or alogliptin.tw.

67. anagliptin/ or anagliptin.tw.

68. dutogliptin/ or dutogliptin.tw.

69. evogliptin/ or evogliptin.tw.

70. gemigliptin/ or gemigliptin.tw.

71. gosogliptin/ or gosogliptin.tw.

72. linagliptin/ or linagliptin.tw.

73. omarigliptin/ or omarigliptin.tw.

74. saxagliptin/ or saxagliptin.tw.

75. septagliptin.tw.

76. sitagliptin/ or sitagliptin.tw.

77. teneligliptin/ or teneligliptin.tw.

78. trelagliptin/ or trelagliptin.tw.

79. vildagliptin/ or vildagliptin.tw.

80. antidiabetic agent/

81. oral antidiabetic agent/

82. or/8-81

83. randomized controlled trial/ or double-blind procedure/ or single-blind procedure/ or (random$ or factorial$ or placebo$ or (double$ adj blind$) or (singl$ adj blind$) or assign$ or allocat$).tw.

84. 7 and 82 and 83

85. MEDLINE.cr.

86. 84 not 85

87. (mouse or mice or murine or rat or rats or dog or dogs or animal*).ti.

88. 86 not 87

89. limit 88 to yr="2015 -Current"

**CENTRAL search**

Date Run: 03/12/2020

ID Search Hits

#1 MeSH descriptor: [Diabetes Mellitus] this term only

#2 ((diabetes or diabetic* or "diabetes mellitus") near/1 ("type 2" or "type ii" or "non-insulin dependent" or "non insulin dependent" or "noninsulin dependent" or "adult onset" or "mature onset" or "late onset")):ti,ab,kw

#3 ("diabetes mellitus" not "insulin dependent diabetes mellitus"):kw

#4 #3 and EMBASE

#5 NIDDM:ti,ab,kw

#6 ((diabetic next nephropath*) or "diabetic kidney disease"):ti,ab,kw

#7 #1 or #2 or #4 or #5 or #6

#8 ((long acting or longer acting or intermediate acting) near/1 insulin*):ti,ab,kw

#9 insulin near/1 degludec:ti,ab,kw

#10 insulin near/1 detemir:ti,ab,kw

#11 insulin near/1 glargine:ti,ab,kw

#12 insulin near/1 zinc

#13 insulin near/1 aspart:ti,ab,kw

#14 insulin near/1 lispro:ti,ab,kw

#15 insulin near/1 isophane:ti,ab,kw

#16 insulin near/1 ultralente:ti,ab,kw

#17 insulin near/1 lente

#18 insulin near/1 glulisine

#19 fiasp:ti,ab,kw

#20 novorapid:ti,ab,kw

#21 meglitinide*:ti,ab,kw

#22 mitiglinide:ti,ab,kw

#23 nateglinide:ti,ab,kw

#24 repaglinide:ti,ab,kw

#25 (amylin next analogue* or amylin next derivative*):ti,ab,kw

#26 pramlintide:ti,ab,kw

#27 biguanide*:ti,ab,kw

#28 metformin:ti,ab,kw

#29 (sulfonylurea* or sulphonylurea*):ti,ab,kw

#30 acetohexamide:ti,ab,kw

#31 carbutamide:ti,ab,kw

#32 chlorpropamide:ti,ab,kw

#33 glibenclamide:ti,ab,kw

#34 gliclazide:ti,ab,kw

#35 glimepiride:ti,ab,kw

#36 glibornuride:ti,ab,kw

#37 glipizide:ti,ab,kw

#38 gliquidone:ti,ab,kw

#39 glyburide:ti,ab,kw

#40 glycopyramide:ti,ab,kw

#41 tolazamide:ti,ab,kw

#42 glisoxepide:ti,ab,kw

#43 sulphonylurea:ti,ab,kw

#44 tolbutamide:ti,ab,kw

#45 tolcyclamide:ti,ab,kw

#46 glyclopyramide:ti,ab,kw

#47 glycyclamide:ti,ab,kw

#48 metahexamide:ti,ab,kw

#49 MeSH descriptor: [alpha-Glucosidases] this term only

#50 alpha next glucosidase next inhibitor*:ti,ab,kw

#51 acarbose:ti,ab,kw

#52 miglitol:ti,ab,kw

#53 voglibose:ti,ab,kw

#54 (glitazone near/1 (derivative* or analogue*)):ti,ab,kw

#55 thiazolidinedione*:ti,ab,kw

#56 pioglitazone:ti,ab,kw

#57 rivoglitazone:ti,ab,kw

#58 rosiglitazone:ti,ab,kw

#59 lobeglitazone:ti,ab,kw

#60 MeSH descriptor: [Receptors, Glucagon] explode all trees and with qualifier(s): [agonists - AG]

#61 MeSH descriptor: [Glucagon-Like Peptide 1] explode all trees and with qualifier(s): [antagonists & inhibitors - AI]

#62 ("glucagon-like peptide 1 receptor inhibitor" or "glucagon-like peptide 1 receptor agonist" or "glucagon-like peptide 1 inhibitor" or "glucagon-like peptide 1 agonist" or "GLP-1 receptor inhibitor" or "GLP-1 receptor agonist" or "GLP-1 inhibitor" or "GLP-1 agonist"):ti,ab,kw

#63 ("glucagon-like peptide 1 receptor inhibitors" or "glucagon-like peptide 1 receptor agonists" or "glucagon-like peptide 1 inhibitors" or "glucagon-like peptide 1 agonists" or "GLP-1 receptor inhibitors" or "GLP-1 receptor agonists" or "GLP-1 inhibitors" or "GLP-1 agonists"):ti,ab,kw

#64 albiglutide:ti,ab,kw

#65 dulaglutide:ti,ab,kw

#66 (exenatide or "exendin 4"):ti,ab,kw

#67 liraglutide:ti,ab,kw

#68 lixisenatide:ti,ab,kw

#69 semaglutide:ti,ab,kw

#70 taspoglutide:ti,ab,kw

#71 ((DPP4 or DPP 4 or DPP IV) next inhibitor*):ti,ab,kw

#72 dipeptidyl-peptidase IV Inhibitor:ti,ab,kw

#73 dipeptidyl-peptidase 4 Inhibitor:ti,ab,kw

#74 dipeptidyl-peptidase IV Inhibitors:ti,ab,kw

#75 dipeptidyl-peptidase 4 Inhibitors:ti,ab,kw

#76 alogliptin:ti,ab,kw

#77 anagliptin:ti,ab,kw

#78 gemigliptin:ti,ab,kw

#79 linagliptin:ti,ab,kw

#80 omarigliptin:ti,ab,kw

#81 saxagliptin:ti,ab,kw

#82 sitagliptin:ti,ab,kw

#83 teneligliptin:ti,ab,kw

#84 vildagliptin:ti,ab,kw

#85 dutogliptin:ti,ab,kw

#86 evogliptin:ti,ab,kw

#87 gosogliptin:ti,ab,kw

#88 septagliptin:ti,ab,kw

#89 trelagliptin:ti,ab,kw

#90 MeSH descriptor: [Sodium-Glucose Transporter 2 Inhibitors] this term only

#91 ("sodium glucose transporter 2 inhibitor" or "sodium glucose transporter ii inhibitor" or "SGLT 2 inhibitor" or "sodium glucose transporter 2 inhibitors" or "sodium glucose transporter ii inhibitors" or "SGLT 2 inhibitors"):ti,ab,kw

#92 ("sodium glucose cotransporter" near/3 inhibitor*):ti,ab,kw

#93 ("sodium glucose co-transporter" near/3 inhibitor*):ti,ab,kw

#94 canagliflozin:ti,ab,kw

#95 dapagliflozin:ti,ab,kw

#96 empagliflozin:ti,ab,kw

#97 ertugliflozin:ti,ab,kw

#98 tofogliflozin:ti,ab,kw

#99 ipragliflozin:ti,ab,kw

#100 luseogliflozin:ti,ab,kw

#101 remogliflozin:ti,ab,kw

#102 sotagliflozin:ti,ab,kw

#103 MeSH descriptor: [Hypoglycemic Agents] this term only

#104 (antidiabetic next agent*):kw

#105 (oral next antidiabetic next agent*):kw

#106 MeSH descriptor: [Insulin] this term only

#107 {OR #8-#106}

#108 #7 and #107 in Trials

#109 #108 with Publication Year from 2015 to 2020, in Trials
